# Non-viable chicken embryos: an overlooked niche harbouring a significant source of multidrug resistant bacteria in the poultry production

**DOI:** 10.1080/23144599.2019.1698145

**Published:** 2020-01-23

**Authors:** Ruwani Karunarathna, Khawaja Ashfaque Ahmed, Mengying Liu, Chenfang Yu, Shelly Popowich, Kalhari Goonewardene, Thushari Gunawardana, Shanika Kurukulasuriya, Ashish Gupta, Lisanework E. Ayalew, Philip Willson, Musangu Ngeleka, Susantha Gomis

**Affiliations:** a Department of Veterinary Pathology, Western College of Veterinary Medicine, University of Saskatchewan, Saskatoon, Canada; b Canadian Centre for Health and Safety in Agriculture, University of Saskatchewan, Saskatoon, Canada; c Department of Veterinary Microbiology, Western College of Veterinary Medicine, University of Saskatchewan, Saskatoon, Canada; d Clinical Microbiology, Prairie Diagnostic Services, Saskatoon, Canada

**Keywords:** Chicken embryos, AMR, multi-drug resistance, antibiotics, hatchery

## Abstract

Antimicrobial resistance (AMR) is a global issue, posing a grave threat to the public, animal, and environmental health. The AMR surveillance at the level of the hatchery is crucial to develop an AMR control strategy in the poultry industry. The objective of this study was to investigate the AMR profiles of bacteria isolated from yolk material of non-viable broiler chicken embryos at hatch from commercial hatcheries in western Canada. Antimicrobial susceptibility testing was done using the Kirby–Bauer disk diffusion method focusing on *Escherichia coli* (n = 170) and *Enterococcus* (n = 256) species, which are commonly used as indicators of AMR evolution. *E. coli* isolates were resistant to tetracycline, ampicillin, amoxycillin-clavulanic acid, triple sulpha, ceftiofur, gentamycin, and spectinomycin at the rate of 52.9%, 50.6%, 40.0% 31.8%, 29.4%, 29.4%, 21.8% respectively. Among those, 37.1% of *E. coli* were multidrug resistant. The descending order of antimicrobial resistance of *E. faecalis* was; tetracycline (61.9%), ceftiofur (46.2%), bacitracin (43.9%), erythromycin (31.4%) and tylosin (27.4%). Multidrug resistance was detected in 40.4% of *E. faecalis* isolates, and 85.7% of *E. faecium* isolates. To the best of our knowledge, this is the first report on AMR surveillance of non-viable chicken embryos. Overall, the present study revealed that non-viable chicken embryos, an overlooked niche for AMR surveillance, harbour multidrug-resistant *E. coli*, and enterococci that can be a substantial source of superbugs in the environment. Our data also highlight the urgency of including non-viable chicken embryos in AMR surveillance programme to understand AMR dissemination and its control.

## 1. Introduction

Antimicrobial resistance (AMR) has become a serious threat to public, animal and environmental health [1,2]. AMR control is a global priority and the World Health Organization (WHO) has initiated a global action plan to mitigate the emergence and dissemination of AMR [1,2].The emergence of AMR is multifactorial and may include indiscriminate antimicrobial use and resistance gene transfer from one organism to another. The inappropriate and excessive antimicrobial use in farm animals has been suggested as one of the major causes of the emergence of multidrug-resistant superbugs [3]. Consumer awareness about the antimicrobial use in farm-animals and the potential of AMR development is dictating a trend of an increased market demand for organic and antibiotics-free animal products [4].

The European Union banned the vancomycin analogue, avoparcin, in 1997 and bacitracin, spiramycin, tylosin, and virginiamycin in 1999 for the purpose of prophylactic antimicrobial use in farm animals including poultry feed [5]. Although a reduction of vancomycin resistant enterococci (VRE) was observed in poultry products in the European Union following the ban on avoparcin since 1997, there has been no reduction of VRE observed in humans [5]. Moreover, the fluoroquinolone ban in the USA since 2006 as therapeutic use in the poultry industry, did not result in the reduction of ciprofloxacin resistant *Campylobacter* in poultry products [6]. Because of these complexities and poor understanding of AMR, concerted efforts are required to identify the potential sources of AMR in a variety of agricultural settings to develop an appropriate control measures [7].

Although, there is no direct evidence available, however literatures suggest that poultry is a potential source of AMR transmission to humans [8]. In commercial poultry production, AMR development and dissemination can occur at several stages of production, such as, at breeder level, at hatchery and at the production farm level. Most of the data on AMR in poultry were generated from the production farms [9] or from the retail poultry meat [10]. In the poultry industry, commercial hatcheries act as a link between breeder farms and the production farms. Recent studies suggest that the hatchery is a potential reservoir for antimicrobial resistant bacteria [2] and day-old chicks are a potential source of AMR in chicken farms [11]. The comparison of AMR data generated from hatchery samples versus AMR data obtained from poultry farms at the end of production cycle may provide important clue regarding AMR development and its dissemination in the poultry industry [12]. The bacterial contamination of hatching eggs can occur at breeder farm level, egg transport and storage, and at hatchery level [13]. Bacterial contamination of developing chicken embryos in hatcheries occurs in many possible ways including contamination of egg shells and penetration of bacteria via cracks in the egg shell, or due to thin egg shells [2,14]. Transmission of bacteria from hatching eggs to their progeny has been demonstrated for bacterial species such as *Campylobacter* and *Salmonella* [15,16]. Most of the studies related to AMR surveillance at the hatchery level have profiled fluff-derived bacteria [17] or day-old chicks [12]. Given that contaminated eggs explode during incubation [18], which may facilitate dissemination of AMR from dead embryos to healthy live embryos and ultimately reaching to humans through contaminated poultry. The contaminated non-viable chicken embryos have been an overlooked niche for AMR surveillance.

Our recent study revealed that the majority of non-viable broiler chicken embryos examined in western Canadian hatcheries were co-infected with *Enterococcus* species and *Escherichia coli* [19]. *Enterococcus* species and *E. coli* colonizing the gut of animals are used as bacterial indicators to monitor the prevalence and dissemination of AMR between food animal species and humans [20]. Moreover, *E. coli* and *Enterococcus* species can cause significant economic loses to the poultry industry [21]. Hence, present study was designed to fill the knowledge gap by investigating AMR of non-viable chicken embryo using clinical microbiology technique [22]. To the best of our knowledge, this is the first report on AMR surveillance on non-viable chicken embryos in hatcheries.

## 2. Materials and methods

### 2.1. Bacterial isolates

*E. coli* (n = 170) and *Enterococcus* (n = 256) isolates *i.e*. [*E. faecalis* (n = 223), *E. faecium (*n = 21), *Enterococcus avium* (n = 5), *Enterococcus gallinarum* (n = 5) and *Enterococcus casseliflavus* (n = 2)] were recovered from yolk material of non-viable broiler chicken embryos at hatch (21 days of incubation), from three commercial broiler hatcheries in western Canada during 2013 and 2014 [19]. Bacterial swabs were cultured on 5% Columbia sheep blood agar (BA) (Oxoid Company, Napean, ON) and bacterial identification was done by matrix assisted laser desorption ionization-time of flight mass spectrometry (MALDI-TOF MS) (Bruker Daltonics, Milton, ON) as previously described [23]. Bacterial isolates were stored in brain heart infusion (BHI) broth (DIFCO®, Detroit, MI) containing 20% glycerol (Thermo Fisher Scientific, Waltham, MA) at −80 C for further studies.

### 2.2. Antimicrobial susceptibility testing

Each bacterial isolate was streaked on 5% Columbia sheep BA and incubated at 37 C overnight and tested for antimicrobial susceptibility testing using the standard Kirby–Bauer disk diffusion method. Selection of disk concentration, test method and interpretation of zone diameter were done as recommended by the Clinical Laboratory Standards Institute (CLSI) [24,25]. *E. coli* (ATCC 25,922) and *Staphylococcus aureus* (ATCC 25,923) were used as reference strains for *E. coli* and *Enterococcus* species respectively. The following antimicrobial agents and disk potency were used: amoxycillin-clavulanic acid (AUG,30 μg), ampicillin (AMP,10 μg), apramycin (APR,15 μg), bacitracin (BAC, 10 IU), ceftiofur (CEF, 30 μg), chloramphenicol (CHL, 30 μg), ciprofloxacin (CIP, 5 μg), enrofloxacin (ENR, 5 μg), erythromycin (ERY, 15 μg), florfenicol (FLO, 30 μg), gentamicin (GEN, 10 μg), gentamicin [(120 μg, to determine high level resistance to aminoglycosides in *Enterococcus* species)], lincomycin (LIN, 2 μg), neomycin (NEO, 30 μg), penicillin G (PEN, 10 units), spectinomycin (SPE, 100 μg), tetracycline (TET, 30 μg), trimethoprim-sulphonamide (SXT, 1.25 μg), triple sulpha (SSS, 0.25 mg) and tylosin (TYL, 60 μg), vancomycin (VAN, 30 μg). The antimicrobials used in this study represented 10 classes; namely β-lactams (AUG, AMP, CEF, PEN), aminoglycosides (GEN, NEO, SPE), cyclic polypeptides (APR, BAC), phenicols (CHL, FLO), fluoroquinolones (CIP, ENR), lincosamides (LIN), macrolides (ERY, TYL), tetracyclines (TET), glycopeptides (VAN) and folate pathways inhibitors (SSS, SXT). The inhibition zone diameter of each antimicrobial agent was measured using the BIOMIC V3 − 2014-Microbiology Digital Image Analysis system (Giles Scientific Inc, Santa Barbara, California, USA). Inhibition zone diameters were used to categorize antimicrobial susceptibility of the isolate as susceptible, intermediate and resistant according to the CLSI recommendations except for sulphonamides, where the European Committee on Antimicrobial Susceptibility Testing (EUCAST) version 4.0 interpretive criteria were used [26]. Multidrug resistance was enumerated as acquired non-susceptibility to at least one agent in three or more antimicrobial classes [27]. Intrinsic AMR was disregarded in this enumeration.

## 3. Results

### 3.1. Antimicrobial resistance of E. coli

**Figure 1.**
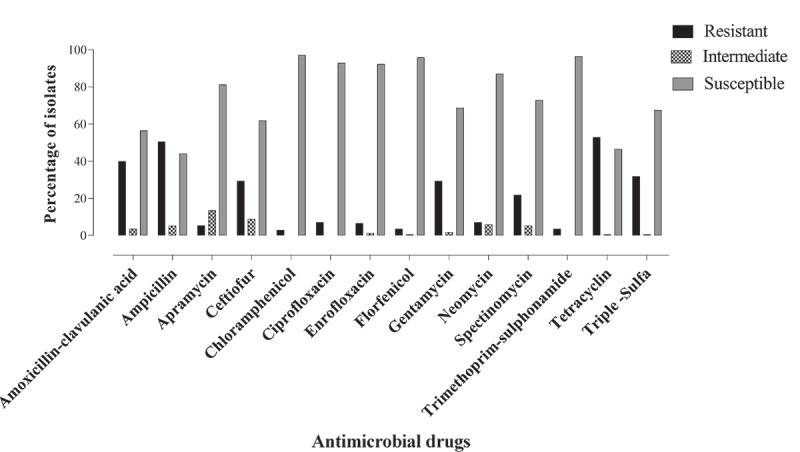
Antimicrobial resistance profile of *E. coli.*

**Figure 2.**
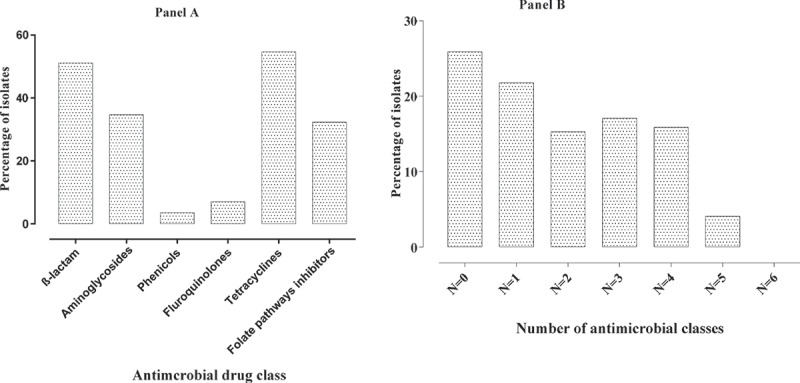
**(Panel A)** Antimicrobial resistance profile of *E. coli* to each drug class and **(panel B)** indicates the multidrug resistance profile of *E. coli.*

*E. coli* isolates were resistant to TET, AMP, AUG, SSS, CEF, GEN and SPE at the rate of 52.9%, 50.6%, 40.0%, 31.8%, 29.4%, 29.4% and 21.8% respectively. The descending order of AMR to the remainder of the antimicrobials were CIP (7.1%), NEO (7.1%), ENR (6.5%), APR (5.3%), FLO (3.5%), SXT (3.5%) and CHL (2.9%) (Figure 1). Multidrug resistance was seen in 63 of 170 (37.1%) *E. coli* isolates of which 17.1% (n = 29) of *E. coli* were resistant to three classes of antimicrobials, 15.9% (n = 27) of *E. coli* were resistant to four classes of antimicrobials and 4.1% (n = 7) of *E. coli* were resistant to five classes of antimicrobials (Figure 2). The intrinsic resistance of *E. coli* was noted for BAC (99.4%), LIN (99.4%), TYL (98.2%), VAN (97.7%), PEN (97.1%) and ERY (91.2%). The AMR profile of all *E. coli* isolates are shown in Tables 1 and 2. AMR phenotypes of *E. coli*, in descending order, were TET (23/170), AUG (R) + AMP(R) + CEF(R) + GEN(R) + SPE(R) + TET(R) + SSS (R) (9/170), AUG (R) + AMP (R) + CEF (R) + CIP (R) + ENR (R) + TET (R) + SSS (R) (8/170) and AUG (R) + AMP (R) + CEF (R) (8/170). Pan-resistance was not observed for *E. coli* but pan-susceptibility was observed in 18.82% isolates.

**Table 1.** Antimicrobial resistance profile of *E. coli.*

Drug class	Drug	Disk potency	Resistance percentage (n = 170)
β-lactam	AUG	30 μg	40.0
	AMP	10 μg	50.6
	CEF	30 μg	29.4
Phenicols	CHL	30 μg	2.9
	FLO	30 μg	3.5
Fluoroquinolones	ENR	5 μg	6.5
	CIP	5 μg	7.1
Aminoglycosides	GEN	10 μg	29.4
	NEO	30 μg	7.1
	SPE	100 μg	21.8
Tetracyclines	TET	30 μg	52.9
Cyclic polypeptides	APR	15 μg	5.3
Folate pathways inhibitors	SSS	31.58 μg	31.8
	SXT	1.25–23.75 μg	3.5

**Table 2.** Summary of resistance profiles of *E. coli* (n = 170)

Resistance profile										Number of isolates
AUG	AMP	CEF	CIP	ENR	GEN	SPE	TET	SXT	SSS	2
AUG	AMP	CEF	CHL	FLO	GEN	NEO	TET	SSS		1
AUG	AMP	CEF	CHL	FLO	GEN	SPE	TET	SSS		1
AUG	AMP	CEF	CIP	GEN	SPE	TET	SSS			1
AUG	AMP	CHL	FLO	GEN	SPE	TET	SSS			1
AUG	CEF	CHL	FLO	GEN	SPE	TET	SSS			1
AUG	AMP	CEF	GEN	SPE	TET	SSS				9
AUG	AMP	CEF	CIP	ENR	TET	SSS				8
AUG	AMP	CEF	CHL	FLO	TET	SSS				1
AUG	AMP	GEN	NEO	TET	SXT	SSS				1
AUG	AMP	APR	CEF	GEN	NEO	TET				1
AUG	AMP	CEF	GEN	SPE	SSS					3
AUG	AMP	GEN	SPE	TET	SSS					3
AUG	AMP	CEF	GEN	TET	SSS					1
AUG	AMP	CEF	CIP	ENR	SSS					1
AUG	AMP	CEF	FLO	TET	SSS					1
AUG	AMP	APR	CEF	GEN	NEO					1
AUG	AMP	APR	CEF	NEO						2
AUG	AMP	CEF	GEN	SSS						1
AUG	AMP	GEN	NEO	TET						1
AUG	AMP	GEN	TET	SSS						1
AUG	AMP	TET	SXT	SSS						1
AUG	AMP	CEF	TET							4
AUG	AMP	CEF	GEN							1
AUG	AMP	CEF	SPE							1
AUG	AMP	GEN	TET							2
AMP	GEN	SPE	TET							2
AUG	AMP	SPE	SSS							1
AUG	AMP	TET	SSS							1
AMP	SPE	TET	SSS							1
APR	GEN	NEO	SPE							1
GEN	SPE	TET	SSS							5
GEN	SPE	TET	SXT							1
AUG	AMP	CEF								8
AUG	AMP	TET								5
GEN	SPE	SSS								3
AMP	GEN	TET								2
NEO	TET	SSS								2
AMP	CEF	GEN								1
AMP	SPE	SSS								1
AMP	SXT	SSS								1
APR	NEO	TET								1
GEN	TET	SSS								1
AMP	TET									5
AUG	AMP									2
AMP	GEN									2
AMP	APR									1
APR	NEO									1
TET										23
AMP										3
APR										1
Pan-susceptible										32
Other (Non-characterized)										12

### 3.2. Antimicrobial resistance of Enterococcus species

**Figure 3.**
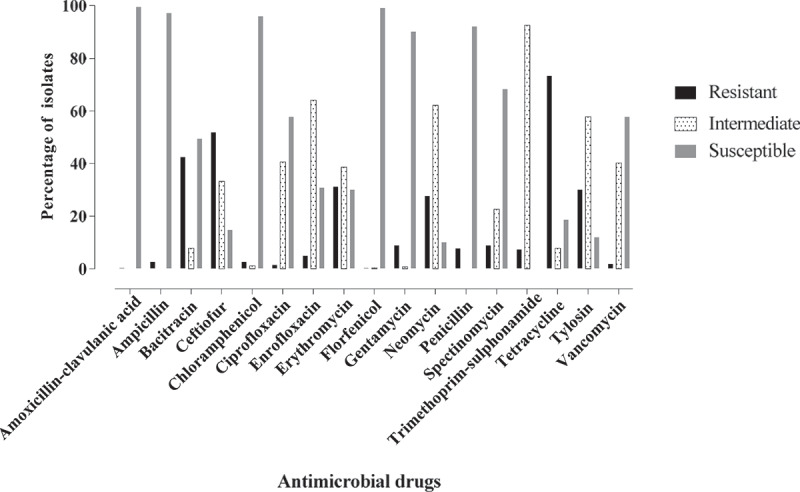
Antimicrobial resistance profile of *Enterococcus* species. The descending order of resistance was seen for tetracycline, ceftiofur, bacitracin, erythromycin and tylosin

**Figure 4.**
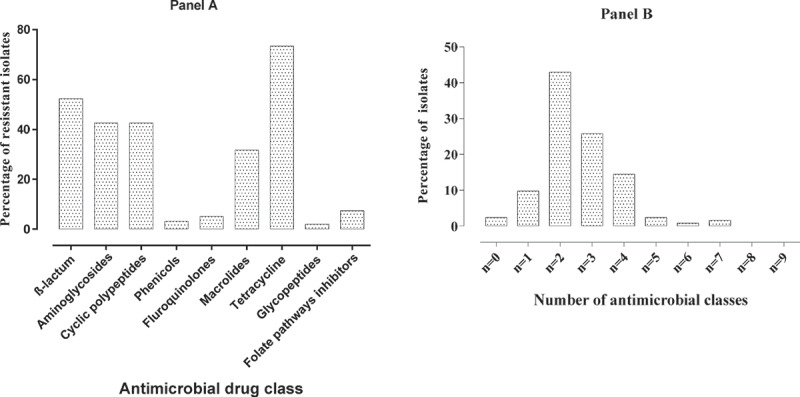
**(Panel A)** Resistant profile of *Enterococcus* species to different classes of antimicrobials and **(Panel B)** Multidrug resistance profile of *Enterococcus* species

All *Enterococcus* isolates were resistant to at least one antimicrobial agent. Antimicrobial resistance phenotypes of *Enterococcus* isolates, in descending order, were TET (73.4%), CEF (51.9%), BAC (42.6%), ERY (31.2%), TYL (30.1), NEO (27.7%), GEN (8.98%), SPE (8.98%), PEN (7.8%), SXT (7.4%), ENR (5.1%), AMP (2.7%), CHL (2.7%), VAN (1.9%), CIP (1.6%), AUG (0.4%) and FLO (0.4%) (Figure 3). Only 3.9% (10/256) of *Enterococcus* isolates were resistant to high concentration of GEN. Multidrug resistance was seen in 44.9% *Enterococcus* isolates of which 25.8%, 14.4%, 2.3%, 0.8% and 1.6% of *Enterococcus* isolates were resistant to three, four, five, six, and seven classes of antimicrobials, respectively (Figure 4). No pan-resistant or pan-susceptible *Enterococcus* isolates were observed. The intrinsic resistance of *Enterococcus* isolates were noted for APR (98.83%) and LIN (96.88%).

**Figure 5.**
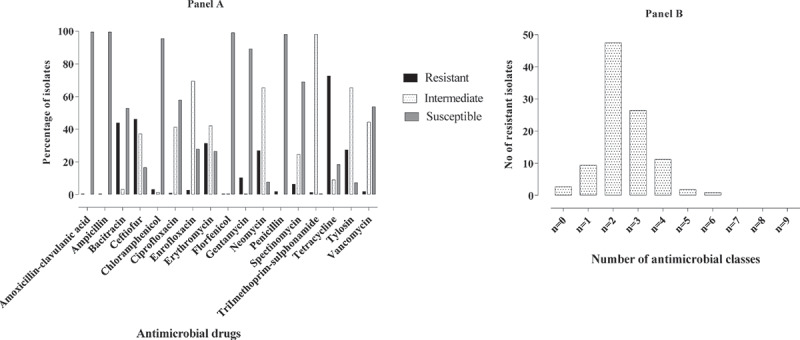
**(Panel A)** Antimicrobial resistance profile of *E. faecalis* and **(Panel B)** indicates Multidrug resistance profile of *E. faecalis.*

AMR profiles of *E. faecalis* and *E. faecium* were summarized in Table 3. The descending order of AMR of *E. faecalis* were; TET (72.6%), CEF (46.2%), BAC (43.9%), ERY (31.4%), TYL (27.4%), NEO (26.9%), GEN (10.3%), SPE (6.3%), CHL (3.1%), SXT (1.3%), VAN (1.8%), PEN (1.8%), ENR (2.7%), CIP (0.9%), AMP (0.4%), AUG (0.4%) and FLO (0.4%) (Figure 5). Only 6.3% (14/223) of *E. faecalis* isolates were resistant to high concentration of GEN. Multidrug resistance was seen in 40.4% of *E. faecalis* isolates of which 26.5% of *E. faecalis* isolates were resistant to three classes of antimicrobials, 11.2% of *E. faecalis* isolates were resistant to four classes of antimicrobials, 1.8% of *E. faecalis isolates were* resistant to five classes of antimicrobials and 0.9% of *E. faecalis* isolates were resistant to six classes of antimicrobials (Figure 5). The resistance profiles of all *E. faecalis* isolates are demonstrated in Table 4. The most common resistance phenotype of *E. fecalis* was TET (R) + BAC (R) (37/223) followed by TET (R) + CEF (R) (23/223), TET (12/223) and TET (R) + ERY (R) + NEO (R) + TYL (R) (12/223).

**Table 3.** Antimicrobial resistance profile of *E. faecalis and E. faecium.*

Drug class	Drug	Disk potency	Resistance percentage	
			*E. faecalis*	*E. faecium*
			*(n = 223)*	*(n = 21)*
β-lactam	AUG	30μg	0.4	0
	AMP	10 μg	0.4	28.6
	PEN	10G	1.8	85.7
	CEF	30 μg	46.2	95.2
Phenicols	CHL	30 μg	3.1	0
	FLO	30 μg	0.4	0
Fluoroquinolones	ENR	5 μg	2.7	42.9
	CIP	5 μg	0.9	14.3
Macrolides	ERY	15 μg	31.4	38.1
	TYL	60 μg	27.4	38.1
Aminoglycosides	GEN	10 μg	10.3	4.8
	NEO	30 μg	26.9	47.6
	SPE	100 μg	6.3	33.3
Tetracyclines	TET	30 μg	72.6	61.9
Folate pathways inhibitors	SXT	1.25–23.75 μg	1.3	66.7
Cyclic polypeptides	BAC	10 IU	43.9	42.9%
Glycopeptides	VAN	30 μg	1.8	0

**Table 4.** Summary of resistance profiles of *E. faecalis* (n = 223)

Resistance profile							Number of isolates
TET	BAC	CEF	ERY	TYL	NEO		1
TET	BAC	CEF	ERY	TYL			2
TET	BAC	CEF	NEO	GEN			1
TET	BAC	CEF	GEN				2
TET	BAC	CEF					6
TET	BAC	GEN					1
TET	BAC	ERY	TYL	GEN			1
TET	BAC	ERY	TYL	NEO			1
TET	BAC	ERY	TYL				6
TET	BAC						37
TET	CEF	ERY	NEO	TYL			8
TET	CEF	ERY	NEO				1
TET	CEF	GEN					5
TET	CEF	NEO					6
TET	CEF						23
TET							12
TET	ERY	TYL					7
TET	ERY						1
TET	ERY	GEN	NEO	TYL			1
TET	ERY	NEO	TYL				12
TET	GEN						8
TET	NEO						6
BAC	CEF	ERY	NEO				2
BAC	CEF	ERY	GEN				1
BAC	CEF	ERY					4
BAC	CEF	NEO					2
BAC	CEF						5
BAC	ERY	NEO	TYL				1
BAC							2
CEF	ERY	TYL	NEO				2
CEF	ERY	TYL					6
CEF	NEO	GEN					1
CEF	NEO						3
CEF							5
ERY	TYL						2
ERY	TYL	NEO					1
AUG	GEN	TET					1
AMP	CEF	ENR	PEN				1
CHL	BAC	ERY	TET	TYL			4
CHL	BAC	ERY	ENR	TET	TYL	NEO	1
CHL	BAC	ERY	ENR	TET	TYL		2
CIP	CEF	ENR	PEN				1
FLO	CEF	GEN	TET	SXT	VAN		1
PEN	CEF	BAC	TET				1
PEN	CEF	CIP	ENR				1
SPE	BAC	NEO	TYL	ERY			1
SPE	BAC	CEF	NEO				5
SPE	BAC	CEF					4
SPE	BAC	NEO					1
SPE	BAC						3
SXT	TET	NEO					1
SXT	TET	NEO	CEF				1
VAN	TYL	TET	NEO	ERY	CEF		1
VAN	BAC	ERY	TET	TYL			1
VAN	CEF						1
Other(Non-characterized)							6

**Figure 6.**
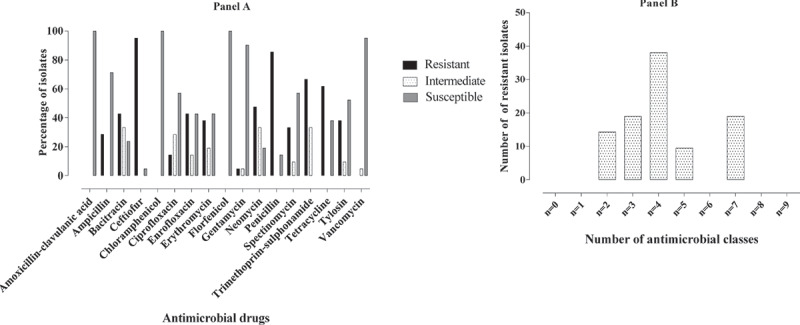
**(Panel A)** Antimicrobial resistance profile of *E. faecium* and **(Panel B)** indicates multidrug resistance profile of *E. faecium.*

The descending order of resistance of *E. faecium* was CEF (95.2%), PEN (85.7%), SXT (66.7%), TET (61.9%), NEO (47.6%), BAC (42.9%), ENR (42.9%), ERY (38.1%), TYL (38.1), SPE (33.3%), AMP (28.6%), CIP (14.3%) and GEN (4.8%). No *E. faecium* was found resistant to AUG, CHL, FLO and VAN (Figure 6). Multidrug resistance was seen in 85.7% of *E. faecium* isolates of which 19.0% of *E. faecium* were resistant to three classes of antimicrobials, 38.1% of *E. faecium* were resistant to four classes of antimicrobials, 9.5% of *E. faecium* were resistant to five classes of antimicrobials and 19.0% of *E. faecium* were resistant to seven classes of antimicrobials (Figure 6). The resistance profiles of all *E. faecium* isolates were shown in Table 5. The most common resistance phenotype was CEF (R) + NEO (R) + TET (R) + SXT (R) + PEN (R) (4/21).

**Table 5.** Summary of resistance profiles of *E. faecium* (n = 21)

Resistance profile											Number of isolates	
CEF	NEO	TET	SXT	PEN								4
AMP	CEF	ENR	PEN	SXT	BAC	ERY	NEO	TET	SPE	TYL		2
AMP	CEF	ENR	PEN	SXT								2
CIP	AMP	BAC	CEF	ENR	ERY	NEO	PEN	SPE	TET	SXT	TYL	1
CIP	ENR	GEN	SXT									1
CIP	AMP	CEF	ENR	ERY	PEN	SXT						1
BAC	CEF	TET	PEN	ENR	ERY	NEO	SPE	SXT	TYL			1
BAC	CEF	TET	PEN	SPE								1
BAC	CEF	TET	PEN	ERY	NEO	TYL						1
BAC	CEF	TET	PEN	SPE								1
BAC	CEF	TET	PEN	ENR	ERY	TYL						1
BAC	CEF											1
CEF	PEN	SXT										1
CEF	SPE	TYL										1
CEF	ERY	PEN	TYL									1
CEF	NEO	TET	SXT	PEN								1

## 4. Discussion

The emergence of AMR is a serious threat to global health, and thus the WHO has recently declared a priority list of pathogens which need novel antibiotic development [28]. Multidrug resistance is a worldwide concern due to failures in treating infectious diseases. The resistance genes are often on mobile genetic elements, including plasmids, integrons, and transposons [29]. The resistance genes are transferred among bacteria via horizontal gene transfer, conjugation, transformation and transduction, which ultimately encodes for multidrug resistance [30]. The present study was designed to investigate the antimicrobial resistance profiles of *E. coli* and *Enterococcus* species isolated from non-viable chicken embryos, an overlooked niche concerning the emergence of multidrug-resistant bacteria.

Our data showed a high degree of resistance of *E. coli* to β-lactam antimicrobials; AMP (50.6%) and AUG (40.0%). Our data in regards to AMP resistance is comparable with AMP resistance of *E. coli* isolated (43%) from poultry products in Canada by the Canadian Integrated Program for Antimicrobial Resistance Surveillance (CIPARS) in 2016 [31]. A recent study has described the emergence of extended-spectrum β-lactamases (ESBLs)-encoding plasmids from *E. coli* isolates in poultry with a similar rate of prevalence as observed in humans which warrants regular monitoring of AMR in the broiler industry [32]. We observed a relatively high prevalence of CEF resistance in *E. coli* (29.4%) which justifies the voluntary withdrawal of this antimicrobial from poultry production in 2014 [31]. It would be interesting to study CEF resistance in *E. coli* from chicken embryo mortality a few years; hence, since CEF resistance of *E. coli* in poultry hatcheries may impose a risk of dissemination to humans. It has been reported that *E. coli* of poultry origin are closely related to *E. coli-*associated extra-intestinal infections in humans [33]. When compared to GEN resistance reported by CIPARS in poultry products (9%), it’s a higher prevalence in *E. coli* isolated in dead embryos [31]. CIPARS represents data of the overall Canadian poultry industry, which may under-represent this emerging ecological milieu in western Canada. However, both Canadian Antimicrobial Resistance Surveillance System (CARSS) and CIPARS have well-documented an increased trend in GEN resistance in *E. coli* isolates of poultry origin during 2004–2014 [34]. GEN is used in the poultry industry to reduce neonatal poultry mortality and for growth promotion [35]. Hence, we can speculate the association of GEN use and increased resistance in the poultry industry in western Canada. In our study, 52.9% of *E.coli* was TET resistant, which is comparable with CIPARS data as they have observed 50% of *E. coli* resistant to TET in 2016 [31]. This trend may be explained by the heavy use of TET in the poultry industry in Canada [36]. There are currently 38 different TET resistance genes described [37], and further investigation is needed to characterize these genes in isolates recovered in our study to determine the resistance mechanisms.

We have seen 1.9% VRE in dead chicken embryos although VAN has not been used in the broiler chicken industry in Canada. The mean VRE increased from 6.2% in 2011 to 7.9% in 2014 in Europe. The frequency of VRE ranged from 0% (Estonia, Finland, Iceland, and Malta) to 45.1% (Ireland). In 2014, increasing trends of VRE were seen in Bulgaria, Croatia, Denmark, Hungary, Ireland, Italy, Slovakia, and United Kingdom from 2011 to 2014 [38]. A study conducted in British Columbia, Canada in 2010 investigating *Enterococcus* isolates obtained from faecal and caecal contents of commercial poultry, demonstrated that none of the enterococci were resistant to VAN [39]. Enterococci of foodborne origin were not identified as a direct cause of resistant enterococci in humans, but they could pose a risk in transfer of resistance determinants to human-adapted strains of the same genus or other genera, as shown in VAN resistance in *S. aureus* and TET and ERY resistance in *Listeria monocytogenes* [40–42].

The resistance of enterococci to TET (73.4%), BAC (42.6%) and TYL (30.1%) was remarkable in our study. It has also been suggested that commensal microbiota of poultry can be a reservoir of BAC resistance, and this BAC resistance can be readily transferable to *E. faecalis* in human [43]. Genes encoding resistance to TET, *tetL* and *tetM*, are frequently associated with *ermB* which encodes resistance to macrolides, lincosamides, streptograminB and quinupristin-dalfopristin. Since BAC is commonly used as a growth-promoting antibiotic in the Canadian poultry industry, resistance to BAC and other antibiotics mentioned above can be co-selected [43]. A recent study conducted in Asia looked at determining AMR of uropathogenic *E. coli* and APEC and found multidrug resistance in 98% of isolates where most of them were resistant to at least five antimicrobials tested [44]. Moreover, emerging extended-spectrum β-lactamases (ESBL) producing *E. coli* were resistant to aminoglycosides and fluoroquinolones [45]. Among them, a classic example of globally disseminated, multidrug-resistant *E. coli* strain sequence type (ST) 131 (ST131) which causes significant amounts of the urinary tract and bloodstream infections in humans [46].

## 5. Conclusion

In the present study, we have observed that chicken embryos harbour a significant number of multidrug-resistant *E. coli* and enterococci, revealing that this niche can be a substantial source of superbugs in the environment. The current antimicrobial resistance surveillance systems predominantly focus on monitoring resistance in poultry farms and processing plants. Embryonated eggs represent a critical niche that can reveal the nature of AMR that would be passed on to the production farms and ultimately to humans via the poultry products. Our data suggest that the screening of antimicrobial resistance, particularly at the level of embryonated eggs, is quintessential in AMR surveillance to understand AMR dissemination for developing appropriate control measures.
